# Identification of disease- and headache-specific mediators and pathways in migraine using blood transcriptomic and metabolomic analysis

**DOI:** 10.1186/s10194-021-01285-9

**Published:** 2021-10-06

**Authors:** Timea Aczél, Tamás Körtési, József Kun, Péter Urbán, Witold Bauer, Róbert Herczeg, Róbert Farkas, Krisztián Kovács, Barna Vásárhelyi, Gellért B. Karvaly, Attila Gyenesei, Bernadett Tuka, János Tajti, László Vécsei, Kata Bölcskei, Zsuzsanna Helyes

**Affiliations:** 1 Department of Pharmacology and Pharmacotherapy, Molecular Pharmacology Research Group and Centre for Neuroscience, University of Pécs Szentágothai Research Centre, University of Pécs Medical School, Szigeti út 12, Pécs, H-7624 Hungary; 2 Department of Neurology, Interdisciplinary Excellence Centre, Faculty of Medicine, Albert Szent-Györgyi Clinical Center, University of Szeged, Semmelweis u. 6, Szeged, H-6725 Hungary; 3 MTA-SZTE Neuroscience Research Group, University of Szeged, Semmelweis u. 6, Szeged, H-6725 Hungary; 4 Faculty of Health Sciences and Social Studies, University of Szeged, Temesvári krt. 31, Szeged, H-6726 Hungary; 5 Szentágothai Research Centre, Bioinformatics Research Group, Genomics and Bioinformatics Core Facility, University of Pécs, Ifjúság útja 20, Pécs, H-7624 Hungary; 6 Department of Laboratory Medicine, Semmelweis University, Nagyvárad tér 4, Budapest, H-1089 Hungary

**Keywords:** Migraine, Transcriptomic analysis, Peripheral blood mononuclear cells, Cytokines, Mitochondrial dysfunction

## Abstract

**Background:**

Recent data suggest that gene expression profiles of peripheral white blood cells can reflect changes in the brain. We aimed to analyze the transcriptome of peripheral blood mononuclear cells (PBMC) and changes of plasma metabolite levels of migraineurs in a self-controlled manner during and between attacks.

**Methods:**

Twenty-four patients with migraine were recruited and blood samples were collected in a headache-free (interictal) period and during headache (ictal) to investigate disease- and headache-specific alterations. Control samples were collected from 13 age- and sex-matched healthy volunteers. RNA was isolated from PBMCs and single-end 75 bp RNA sequencing was performed using Illumina NextSeq 550 instrument followed by gene-level differential expression analysis. Functional analysis was carried out on information related to the role of genes, such as signaling pathways and biological processes. Plasma metabolomic measurement was performed with the Biocrates MxP Quant 500 Kit.

**Results:**

We identified 144 differentially-expressed genes in PBMCs between headache and headache-free samples and 163 between symptom-free patients and controls. Network analysis revealed that enriched pathways included inflammation, cytokine activity and mitochondrial dysfunction in both headache and headache-free samples compared to controls. Plasma lactate, succinate and methionine sulfoxide levels were higher in migraineurs while spermine, spermidine and aconitate were decreased during attacks.

**Conclusions:**

It is concluded that enhanced inflammatory and immune cell activity, and oxidative stress can play a role in migraine susceptibility and headache generation.

**Supplementary Information:**

The online version contains supplementary material available at 10.1186/s10194-021-01285-9.

## Background

Migraine is a primary headache condition characterized by moderate-to-severe unilateral pain of pulsating or throbbing quality and accompanying symptoms, such as nausea/vomiting or photo−/phonophobia. The headache can be triggered by a variety of factors such as alcohol, stress or hormonal changes [1]. There has been an ongoing debate about the precise pathophysiological mechanism of the disease, but the most accepted theory is that migraine is a disorder affecting the sensory processing of the brain [1]. However, the headache is most likely to be generated by the activation of the trigeminovascular system resulting in neurogenic vasodilation and inflammation of the meninges [2]. It is now evident that major contributors to headache development are the neuropeptides calcitonin gene-related peptide (CGRP) [3–5] and pituitary adenylate cyclase activating polypeptide (PACAP) [6–8]. Yet, the exact sequence of events during the phases of headache episode and the relative importance of central and peripheral mechanisms are still unclear. Except for the recently approved anti-CGRP monoclonal antibodies, most of the preventive treatment is based on empirical observations rather than the understanding of the pathophysiology. Elucidating the pathophysiological mechanisms is crucial to identify the key mediators and determine novel therapeutic targets.

It is also accepted that migraine susceptibility has a genetic background [9, 10]. However, most of the research using linkage, candidate gene- and genome-wide association studies (GWAS) provided limited results. GWAS have revealed susceptibility genes or loci implicating vascular and smooth muscle tissues, synaptic function, astrocyte-, microglia- and oligodendrocyte roles [11–13]. The few genomic next-generation sequencing studies mainly focused on certain candidate genes associated with glutamatergic neurotransmission and synaptic function/development, pain-sensing mechanisms, metalloproteinases and vascular metabolism [9]. Quite recently, the interaction between single nucleotide polymorphisms of three genes involved in synaptic transmission was also linked to migraine susceptibility [14]. None of the candidate genes could be conclusive as genetic biomarkers of the disease, each having small impact individually and limited predictive value [15]. The reason for this could be that genetic-environmental interactions play an important role in disease mechanisms in specific clinical conditions. Gene expression patterns associated with migraine reflect genetic and non-genetic effects and may inform of migraine susceptibility and outcome [16].

Recent results have pointed out that interactions between external stimuli and brain pathological processes may be reflected in peripheral tissues, such as the blood, which facilitates clinical research of several central nervous system diseases without invasive tissue sampling [16–18]. Differentially expressed genes have been described in peripheral whole blood of migraineurs by microarray or bead array [19, 20]. More recently, whole blood next-generation RNA sequencing studies were also performed to compare healthy individuals and migraineurs. While the study by Gerring and coworkers revealed significant changes in immune function and cytokine signaling [21], another study by Kogelman and coworkers reported largely negative results [22].

To unveil pathways responsible for headache generation, a self-controlled study design to compare samples of headache (ictal) and headache-free (interictal) periods is also necessary. Since previous data show that the transcriptome of mononuclear blood cells is more closely correlated with the transcriptome of brain samples [18], RNA sequencing was performed from separated peripheral blood mononuclear cells (PBMCs) instead of the whole blood. We have also complemented the transcriptome analysis of peripheral blood mononuclear cells with a plasma metabolome analysis from simultaneously taken samples.

## Methods

### Study design

The study was approved by the National Public Health Center, Ministry of Human Capacities of Hungary (28324–5/2019/EÜIG). All study participants gave their written informed consent in accordance with the Declaration of Helsinki.

Episodic migraine patients (with or without aura) between the age of 20–65 years were included in the study. Migraineurs were selected in accordance with the criteria of the third edition of International Classification of Headache Disorders [23]: recurrent unilateral, pulsating headache, which manifests in moderate or severe intensity attacks lasting 4–72 h. Headache was aggravated by routine physical activity and associated with nausea and/or vomiting, as well as photophobia and phonophobia. Exclusion criteria for enrolment included chronic inflammatory diseases and depression.

Blood samples were drawn from migraine sufferers in an attack-free period and during an attack. The attack-free (interictal) sample was collected if the patient had no headache for at least 24 h. For ictal samples, affected patients were asked not to start their usual attack treatment until the blood had been taken. There were no restrictions as regards food and drink intake. A detailed questionnaire was used to compile a homogeneous group of migraineurs concerning the features of their disease. Questions included the prophylactic or attack medication before sampling, number of attacks in the previous month, the time of the last attack, the beginning of the current attack, other known diseases, applied drugs and contraceptives, relation of migraine attacks to the menstrual cycle, the presence of allodynia, attack frequency, duration of migraine, severity of pain during attacks as measured on a visual analog scale, co-morbidities with other chronic diseases, familial manifestation of migraine, regular sport activity and the time of the last meal were recorded.

Enrolment took place between September 2018 and December 2019. Thirty six female and 1 male subjects were recruited: 24 episodic migraine patients with or without aura and 13 healthy controls. Sample size was determined based on literature data [24, 25]. Healthy volunteers serving as controls were screened for non-reported/non-treated headaches.

### Sample collection

Human blood (13 mL/person) was collected from cubital veins of migraineurs and healthy volunteers into ice-cold glass tubes containing ethylenediaminetetraacetic acid (EDTA) or citrate. For the transcriptomic measurements, the PBMCs were isolated by Ficoll-Paque PREMIUM (GE Healthcare, Budapest, Hungary) according to the manufacturer’s instructions. Four mL of anticoagulant-treated blood and 4 mL phosphate-buffered saline (PBS) - EDTA solution were mixed in sterile centrifuge tubes. Next, the diluted blood samples were layered on 5 mL Ficoll-Paque PREMIUM and centrifuged 40 min at 400×g, 20 °C. After the removal of the liquid phase, the PBMC layer was transferred into a new centrifuge tube, suspended with 6 mL PBS-EDTA solution and centrifuged 10 min at 500×g, 20 °C. The supernatant was removed also and the pellet was suspended in 6 mL PBS-EDTA solution, followed by centrifugation (10 min, 500×g, 20 °C). Liquid phase was removed and the cells were resuspended with 1 mL of TRI Reagent (Molecular Research Center, Cincinnati, OH, USA), transposed to Eppendorf tubes and stored at − 80 °C until the gene expression investigations.

For the metabolomic measurements, the total human blood samples were centrifuged at 300×g for 15 min, twice at 2500×g for 15 min and 18,000×g for 90 min at 4 °C. Plasma samples were stored at − 80 °C until analysis. Samples showing signs of hemolysis were excluded.

### RNA extraction and quality control

Isolation and purification of total RNA were carried out as previously described [26] using the phenol-chloroform based TRI Reagent procedure (Molecular Research Center, Cincinnati, OH, USA), up to the step of acquiring the RNA-containing aqueous layer. The aqueous phase was mixed with an equal volume of absolute ethanol and was loaded into Zymo-Spin™ IICR Column. Direct-zol RNA MiniPrep kit (Zymo Research, Irvine, CA, USA) was used according to the manufacturer’s protocol including the optional on-column DNase digestion.

RNA concentrations were measured using Qubit 3.0 (Invitrogen, Carlsbad, CA, USA). The RNA quality was verified on TapeStation 4200 using RNA ScreenTape (Agilent Technologies, Santa Clara, CA, USA). We proceeded with high quality (RIN > 8) RNA samples to library preparation.

### Illumina library preparation and sequencing

The library for Illumina sequencing was prepared using NEBNext Ultra II Directional RNA Library Prep Kit for Illumina (NEB, Ipswitch, MA, USA). Briefly, mRNA was isolated from 500 ng total RNA using NEBNext Poly(A) mRNA MAgnetic Isolation Module (NEB, Ipswitch, MA, USA). Thereafter, the mRNA was fragmented, end prepped and adapter-ligated. Finally, the library was amplified according to the manufacturer’s instructions. The quality of the libraries was checked on 4200 TapeSation System using D1000 Screen Tape, the quantity was measured on Qubit 3.0. Illumina sequencing was performed on the NextSeq550 instrument (Illumina, San Diego, CA, USA) with 1 × 76 run configuration.

### Bioinformatics

The sequencing reads were aligned against the *Homo sapiens* reference genome (GRCh37 Ensembl release) with STAR v2.5.3a [27]. After alignment, the reads were associated with known protein-coding genes and the number of reads aligned within each gene was counted using Rsubread package v2.0.0 [28]. Gene count data were normalized using the trimmed mean of M values (TMM) normalization method of the edgeR R/Bioconductor package (v3.28, R v3.6.0, Bioconductor v3.9) [29]. For statistical testing the data were further log transformed using the voom approach [30] in the limma package [31]. Normalized counts were represented as transcripts per million (TPM) values. Fold change (FC) values between the compared groups resulting from linear modeling process and modified t-test *p*-values were produced by the limma package. The Benjamini–Hochberg method was used to control the False Discovery Rate (FDR) and adjusted *p*-values were calculated by limma. In case of paired ictal and interictal samples the correlation between samples originating from the same patient was taken into account using the duplicateCorrelation function of limma. Functional analysis was performed to take into account the annotations of genes using the Gene Ontology (GO), Kyoto Encyclopedia of Genes and Genomes (KEGG), and Reactome databases. Detection of functional enrichment was performed in the differentially expressed gene list (DE list enrichment: Fisher’s exact test for GO, hypergeometric test for KEGG and Reactome) and towards the top of the list when all genes have been ranked according to the evidence for being differentially expressed (ranked list enrichment: non-parametric Kolmogorov-Smirnov test for GO and KEGG, hypergeometric test for Reactome) applying the topGO v2.37.0, ReactomePA v1.30.0, gage v2.36.0 packages. The pathview package v1.26.0 [32] was used to visualize mapping data to KEGG pathways.

### Targeted metabolomic measurements

Acetonitrile, formic acid, methanol and water, all LC-MS grade, as well as ammonium acetate for HPLC and ethanol 96% Ph. Eur. 9.0, were obtained from Molar Chemicals Kft. (Halásztelek, Hungary). The MxP Quant 500 Kit was purchased from Biocrates Life Sciences AG (Innsbruck, Austria). Phenyl isothiocyanate (PITC), phosphate buffered saline and pyridine were from Sigma Aldrich Kft (Budapest, Hungary). Phosphate buffered saline solution was prepared as per the recommendations of the manufacturer. 5 mmol/L ammonium acetate was prepared by adding 19 mg ammonium acetate to 50 mL methanol.

Plasma samples were processed for analysis as recommended by the kit manufacturer. Briefly, after being allowed to thaw and equilibrate to room temperature, samples were homogenized. 10-μL plasma aliquots, calibrators and controls were pipetted into the respective slots of a 96-well deep well reaction plate. The plate was dried for 30 min under nitrogen 5.0 (Messer Hungarogáz Kft., Budapest, Hungary). Derivatization was performed by adding 50 μL 5% PITC prepared in a mixture of ethanol, pyridine and water (1:1:1, v/v) to each slot, covering and incubating the plate for 60 min at ambient temperature and, after removing the plastic lid, by drying for 60 min under nitrogen. 300 μL 5 mmol/L ammonium acetate was subsequently added and the plate was shaken on an Allsheng MD-200 plate shaker at 450 rpm, ambient temperature, for 30 min. Elution of the analytes into a 96-well deep-well collection plate was performed by applying positive pressure on a Phenomenex Presston manifold (Gen-Lab Kft., Budapest, Hungary). For runs including chromatographic separation, 150 μL extract was pipetted to an LC collection plate and was diluted with 150 μL water. For flow injection analysis, 10 μl extract was transferred to a FIA collection plate and was diluted with 490 μL mobile phase employed for the FIA runs.

Analysis was conducted on a Shimadzu Nexera XR high performance liquid chromatograph (Simkon Kft, Budapest, Hungary) coupled to a low-resolution Sciex Qtrap 5500 mass spectrometer equipped with an electrospray ionization unit and operated in the multiple reaction monitoring mode (Per-form Hungária Kft, Budapest, Hungary). Sciex Analyst v.1.6.3 software was used for instrument control and data acquisition. Peak review and analyte quantitation was done using the Biocrates MetIDQ^TM^ (Nitrogen version) software as instructed by the kit manufacturer.

Samples were run using 4 different instrumental setups, with the liquid chromatographic separation of 106 metabolites, and the flow injection analysis of 524 metabolites. Both positive and negative ionization polarity was employed. Liquid chromatographic separation was performed using the stationary phase provided by the kit manufacturer and equipped with a precolumn Mixer (Biocrates A.G., Innsbruck, Austria). The mobile phases were water (A) and acetonitrile (B), both of which contained 0.2% formic acid. Analysis with positive ionization was carried out with an initial flow rate was 0.5 mL/min, then 0.6 mL/min at 5.5 min, then 8.0 mL/min at 7.0 min, and, finally, 0.5 mL/min at 7.5 min. The following linear gradient program was applied (% mobile phase B): initial, 0% for 0.25 min, 12% at 1 min, 17.5% at 3,0 min, 50% at 4.5 min, and 100% at 5.5 min. Analysis in the negative ionization mode was carried out with an initial flow rate of 0.5 mL/min, 0.7 mL/min at 4.5 min, 0.8 mL/min at 6.5 min, and, finally, 0.5 mL/min at 7.6 min. The following linear gradient program was applied: initial, 0% for 0.25 min, 25% at 0.5 min, 50% at 3.0 min, 75% at 4.0 min, and 100% at 4.5 min. The injection volume was 5 μL. The stationary phase was thermostatted at 50 °C. The general mass spectrometry settings in the positive and negative modes, respectively, were curtain gas, 45 L/min and 20 L/min, collision gas, 9 L/min and 8 L/min, ion spray voltage, 5500 V and − 4500 V, ion source temperature, 500 °C and 650 °C, ion source gas 1, 60 L/min and 40 L/min, and ion source gas 2, 70 L/min and 40 L/min. In the flow injection analysis mode, the mobile phase was prepared by adding 1 ampule FIA Mobile Phase Additive, supplied with the MxP Quant 500 kit, to 290 μL methanol. The flow rate was 0.2 mL/min, the sample injection volume was 20 μL. Ionization was performed in the positive mode. In the 2 runs, respectively, curtain gas was 20 L/min and 10 L/min, collision gas was 9 L/min, ion spray voltage was 5500 V, ion source temperature was 200 °C and 350 °C, ion source gas 1 was set at 40 L/min and 30 L/min, and ion source gas 2 was at 50 L/min and 90 L/min. Analyte-specific mass spectrometry settings were provided by the kit manufacturer.

### Evaluation and statistical analysis of targeted metabolomic measurements

The calculation of the concentrations of the metabolites evaluated in the targeted metabolomic measurements, as well as quality control assessment, was performed automatically by the Biocrates MetIDQ^TM^ software. 42 metabolites, all determined in the liquid chromatography-mass spectrometry (LC-MS/MS) assay, were quantitated using 6-point calibration curves. Linear regression was applied using 1/concentration weights, except for dopamine (quadratic regression, 1/concentration weights). The determination coefficients of the fitted lines ranged between 0.9894–0.9999 (median: 0.9972). 64 metabolites, assayed using LC-MS/MS, were evaluated by comparing their peak areas to those of their respective internal standards dried onto each slot of the sample preparation plate in known concentrations. The quantitation of the 524 metabolites measured using flow injection analysis-tandem mass spectrometry (FIA-MS/MS) was performed automatically by the MetIDQ^TM^ software employing algorithms not disclosed to the users of the Biocrates MxP® Quant 500 kit. No data filtering or correction was applied in this phase of evaluation.

Raw metabolomic data treatment included cleaning of the background noise and unrelated ions through Molecular Feature Extraction (MFE) tool in Mass Hunter Qualitative Analysis Software (B.06.00, Agilent). Mass Profiler Professional (B.12.61, Agilent Technologies) software was used to perform quality assurance (QA) procedure and data filtration. QA procedure covered selection of metabolic features with good repeatability. To achieve this, only features detected in > 80% of the samples after QC, and samples having RSD < 30% were kept.

The differences between metabolomic profiles of the healthy controls and migraineurs (interictal and ictal) patients were studied. Homogeneity of variance and normality assumptions were studied using Levene’s and Shapiro-Wilk tests respectively. Mean plasma concentrations of metabolites in 3 study groups were compared using one-way analysis of variance (ANOVA) test or Kruskal–Wallis test. For one-by-one comparisons, the t-test or Wilcoxon test were used. The statistical significance level was set at 0.05 for all two-sided tests and multivariate comparisons. All calculations were prepared in R (R version 3.6.2).

## Results

### Clinical characteristics of the patient population

The baseline demographic and clinical characteristic of the studied population is presented in Table 1. The studied groups were well matched, without any between-group differences in age, and anthropometric measurements such as body mass index (BMI). Interictal blood samples were collected from all 24 migraine patients, while ictal samples were obtained from 8 of them for self-controlled comparison.

**Table 1:** Demographic and clinical characteristics of study participants. Mean ± SD values are represented in the table

Group	Migraineurs with (***n*** = 3) and without aura (***n*** = 21)	Healthy control subjects (***n*** = 13)
Gender	female *n* = 23 male *n* = 1	female *n* = 13
Age (years)	35 ± 12.25	35 ± 4.96
Body mass index (BMI)	22.21 ± 4.57	24 ± 3.47
Last meal (hours ago)	6.59 ± 6.29	3.69 ± 5.32
**Co-morbidities and drugs of migraine patients**		
Known other diseases	yes *n* = 10 no *n* = 14	
Regular medication (except for attack therapy)	yes *n* = 7 no *n* = 17	
Hormonal contraceptives	yes *n* = 8 no *n* = 16	
Antimigraine prophylactic therapy	no *n* = 24	
**Clinical features of the headache**		
Disease duration (years)	15 ± 12	
Attack frequency (attack/year)	32 ± 37.37	
Visual analogue scale (VAS)	7 ± 1.44	
Allodynia	yes *n* = 9 no *n* = 15	
Chronic pain	yes *n* = 3 no *n* = 21	
Menstruation-headache relationship	sensitive *n* = 10 independent *n* = 13	
Migraineurs in the family	yes *n* = 15 no *n* = 9	
Regular sport activity	yes *n* = 13 no *n* = 11	
**Features of attacks before samplings**		
Number of attacks in the previous month	3 ± 3.31	
Last attack before interictal blood sampling (days ago)	16.58 ± 28.35	
Beginning of attack before ictal blood sampling (hours)	17.91 ± 29.47	

### Transcriptome profile of PBMC samples

Twenty out of 24 interictal blood samples were used for PBMC RNA sequencing.

In interictal PBMC samples compared to healthy ones, 163 genes were found to be differentially expressed with a fold change threshold of 1.5 and a *p*-value threshold of 0.05, 135 genes were upregulated and 28 were downregulated. Based on the average of fold change and *p*-value ranks (average rank), the interleukin (IL)-1β gene (IL1B) was implicated at the top of the differentially expressed (DE) gene list (Table S1). Other highly implicated genes include prostaglandin-endoperoxide synthase 2 (PTGS2) also known as cyclooxygenase 2 (COX2), tumor necrosis factor (TNF), and numerous chemokines, such as IL-8 (IL8).

In ictal PBMC samples, when compared to the interictal ones, 144 genes were differentially expressed (fold change: 1.3, *p*-value: 0.05), 64 were upregulated, 80 downregulated. Heterogeneous nuclear ribonucleoprotein C like 1 (HNRNPCL1) was implicated most, along with olfactory receptor family 10 subfamily G member 2 (OR10G2) and interleukin 20 receptor subunit alpha (IL20RA), among others (Table S2). After FDR correction, two genes had adjusted *p*-values below 0.25, heterogeneous nuclear ribonucleoprotein C like 1 (HNRNPCL1) and cornichon family AMPA receptor auxiliary protein 3 (CNIH3).

In ictal PBMC samples compared to healthy samples, 131 genes were differentially expressed (fold change: 1.5, *p*-value: 0.05), 118 were upregulated, 13 downregulated. Similarly to the interictal and healthy sample comparison, IL1B gene was implicated at the top of the differentially expressed gene list (Table S3). Other highly implicated genes include PTGS2, TNF, and numerous chemokines such as IL8.

**Fig. 1:**
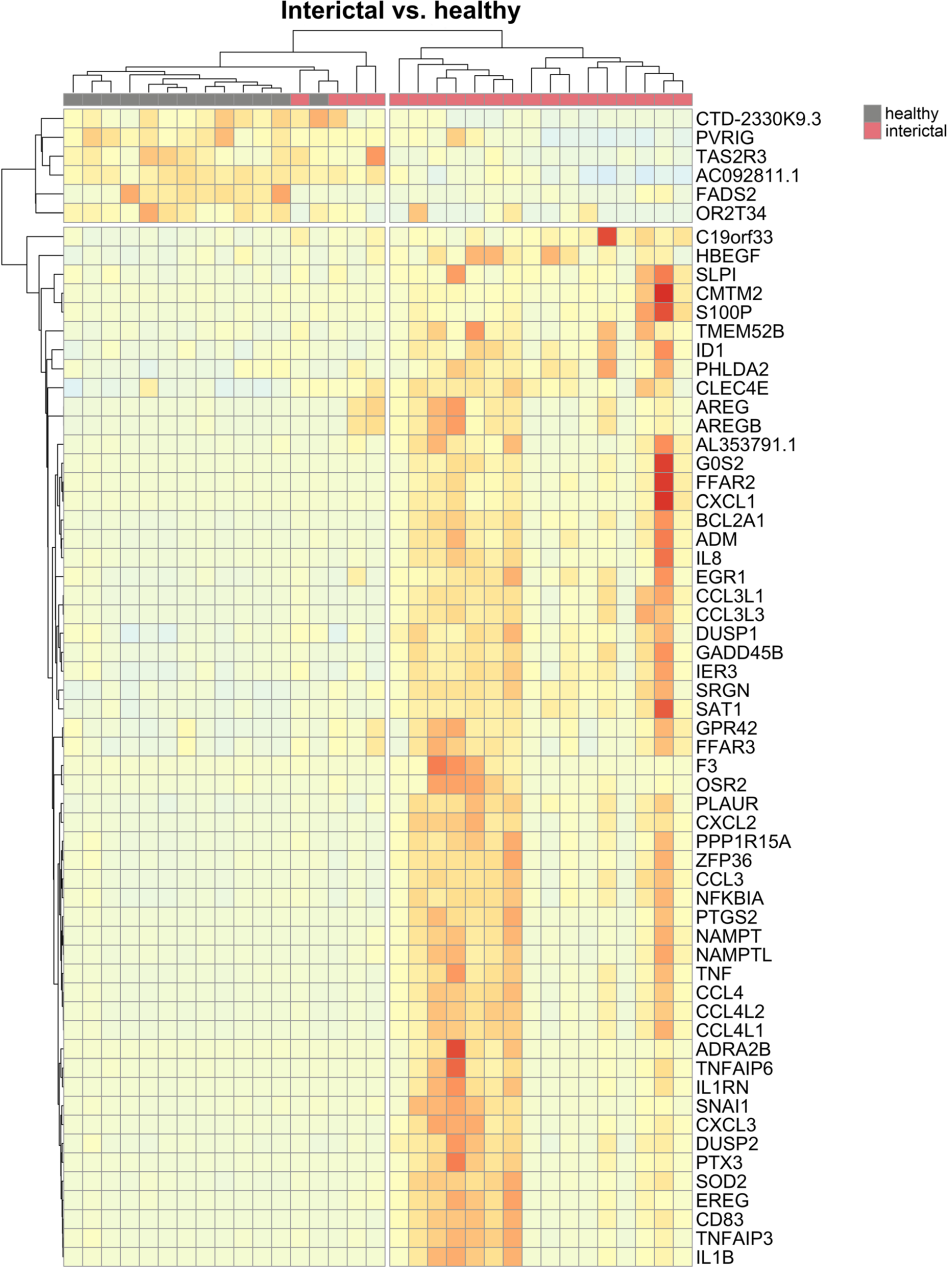
Heat map representation of differentially expressed genes in the interictal vs. healthy PBMC comparison. Columns represent samples, rows represent genes. Pearson correlation was respectively calculated between samples and genes, visualized by dendrograms

**Fig. 2:**
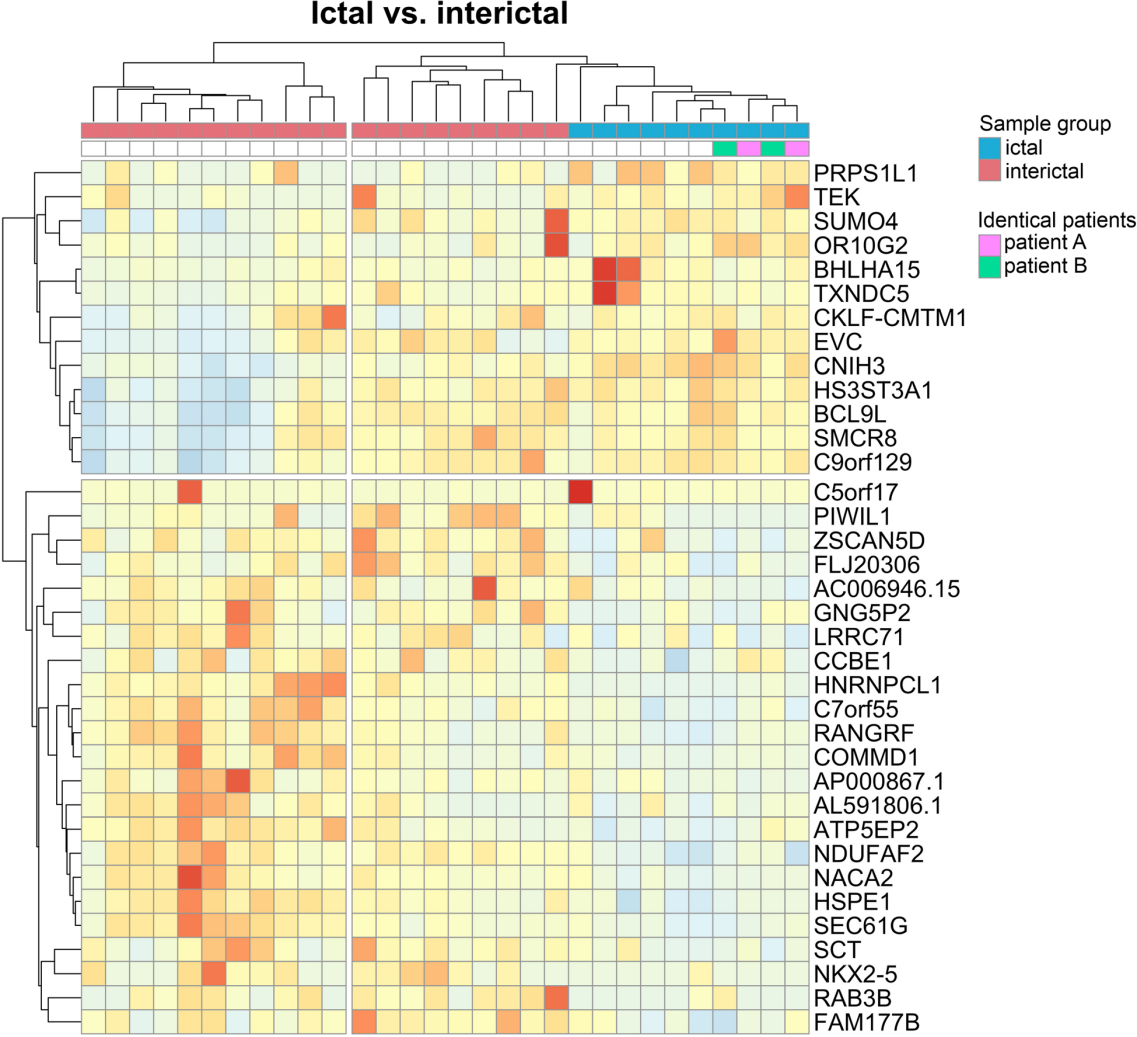
Heat map representation of differentially expressed genes in the ictal vs. interictal comparison. Columns represent samples, rows represent genes. Pearson correlation was respectively calculated between samples and genes, visualized by dendrograms. Ictal samples from identical patients (“**A**”, “**B**”) are marked with respective colors in the legend

**Fig. 3:**
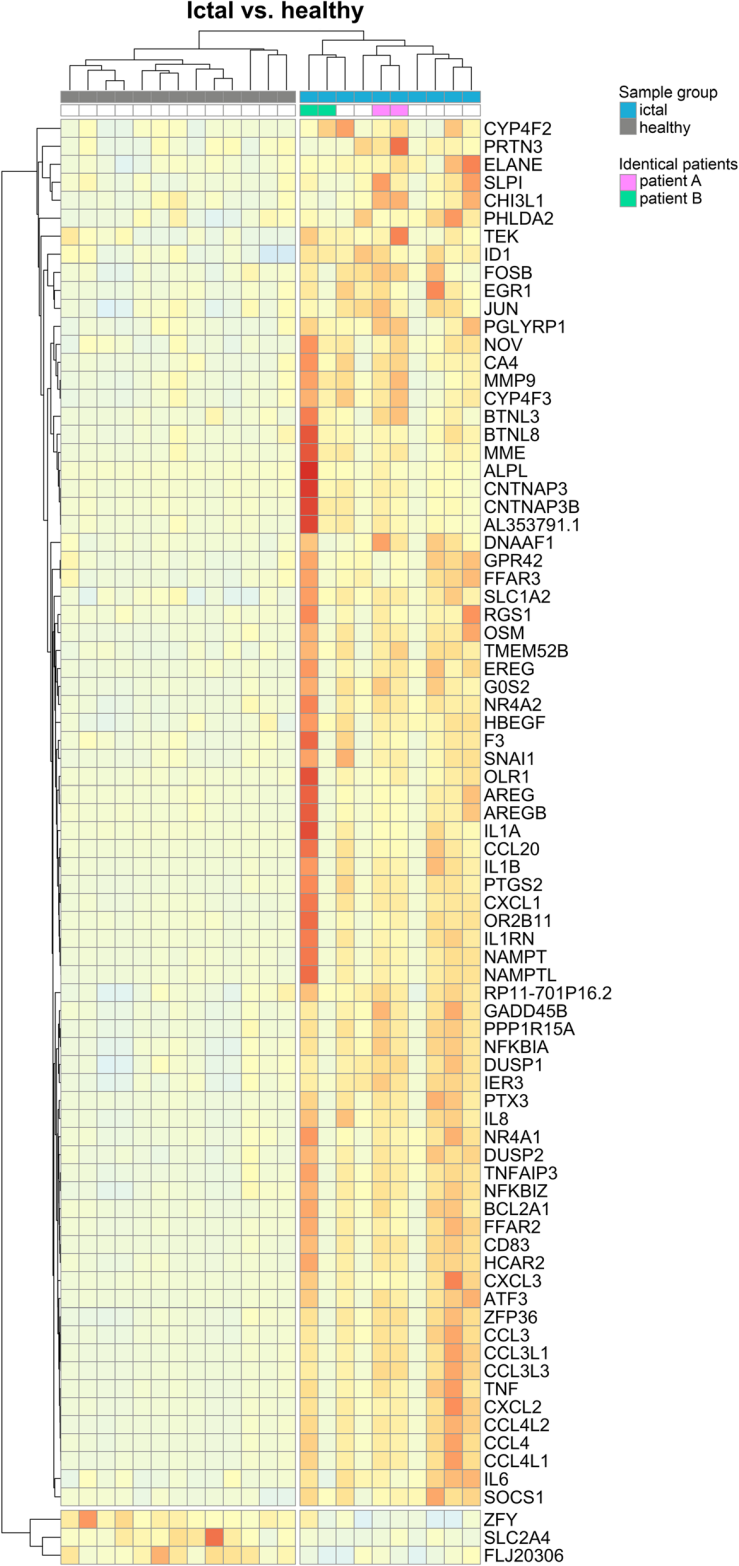
Heat map representation of differentially expressed genes in the ictal vs. healthy comparison. Columns represent samples, rows represent genes. Pearson correlation was respectively calculated between samples and genes, visualized by dendrograms. Ictal samples from identical patients (“**A**”, “**B**”) are marked with respective colors in the legend

When DE genes were visualized on heat maps (Figs. 1, 2, 3), samples were clustered according to the original sample groups. Remarkably, the interictal group splits into two major and one minor subgroups based on gene expression patterns when compared to the healthy (Fig. 1) and ictal (Fig. 2) group.

Functional enrichment analysis of DE genes (DE list enrichment) and ranked list enrichment of all genes were carried out, which yielded statistically significant GO, KEGG and Reactome terms involved in PBMC cells of migraineurs (Tables 2, 3, 4).

**Table 2:** Functional enrichment results of PBMC RNA-seq data of the interictal vs. healthy comparison. DE list enrichment: overrepresentation of functional terms in the differentially expressed gene list. Ranked list enrichment: enrichment of genes associated with a certain pathway towards the top of the ranked whole data. GO: gene ontology, BP: biological process, CC: cellular component, MF molecular function. KEGG: Kyoto Encyclopedia of Genes and Genomes. Annotated: number of genes associated with the given term. R: Reactome database. Significant: number of statistically significant associated with the given term. Expected: expected number of genes associated with the given term in the DE gene list. Gene ratio: ratio of number of genes in the DE list that overlap with genes associated with the given term, and the number of genes in the DE list which overlap with genes associated with all terms in the Reactome database. Bg ratio: ratio of the number of genes associated with the given term, and the total number of genes associated with Reactome terms

Interictal vs. healthy					
ID	DE list enrichment	Annotated	Significant	Expected	*p*-value
GO:0006954 BP	inflammatory response	625	31	6.03	2.60E-14
GO:0005126 MF	cytokine receptor binding	210	14	1.89	6.10E-09
GO:0042379 MF	chemokine receptor binding	37	6	0.33	8.90E-07
		GeneRatio	BgRatio		p-value
R-HSA-6783783	Interleukin-10 signaling	11/96	40/8821		2.60E-14
	Ranked list enrichment	Annotated			p-value
GO:0022900 BP	electron transport chain	173			2.60E-14
GO:0005746 CC	mitochondrial respiratory chain	82			2.17E-05
GO:0016684 MF	oxidoreductase activity, acting on peroxide as acceptor	50			0.000016
KEGG 190	Oxidative phosphorylation	121			8.82E-06
KEGG 4080	Neuroactive ligand-receptor interaction	157			2.28E-05

**Table 3:** Functional enrichment results of PBMC RNA-seq data of the ictal vs. interictal comparison. DE list enrichment: overrepresentation of functional terms in the differentially expressed gene list. Ranked list enrichment: enrichment of genes associated with a certain pathway towards the top of the ranked whole data. GO: gene ontology, BP: biological process, CC: cellular component, MF molecular function. KEGG: Kyoto Encyclopedia of Genes and Genomes. Annotated: number of genes associated with the given term. R: Reactome database. Significant: number of statistically significant associated with the given term. Expected: expected number of genes associated with the given term in the DE gene list. Gene ratio: ratio of number of genes in the DE list that overlap with genes associated with the given term, and the number of genes in the DE list which overlap with genes associated with all terms in the Reactome database. Bg ratio: ratio of the number of genes associated with the given term, and the total number of genes associated with Reactome terms

Ictal vs. interictal					
ID	DE list enrichment	Annotated	Significant	Expected	*p*-value
GO:1902305 BP	regulation of sodium ion transmembrane transport	55	4	0.39	2.60E-14
GO:0070382 CC	exocytic vesicle	175	6	1.3	0.0019
GO:0005179	hormone activity	61	3	0.43	0.00924
KEGG 4742	Taste transduction	35	2	0.219161	0.019843
	Ranked list enrichment	Annotated			p-value
GO:0005746 CC	mitochondrial respiratory chain	82			2.60E-14
GO:0005125 MF	cytokine activity	149			0.000001
GO:0005179 MF	hormone activity	61			6.95E-05
GO:0030594 MF	neurotransmitter receptor activity	57			0.000107
GO:0004984 MF	olfactory receptor activity	44			0.000433
KEGG 4080	Neuroactive ligand-receptor interaction	157			9.04E-05
KEGG 190	Oxidative phosphorylation	121			0.000845
KEGG 140	Steroid hormone biosynthesis	36			0.00112

**Table 4:** Functional enrichment results of PBMC RNA-seq data of the ictal vs. healthy comparison. DE list enrichment: overrepresentation of functional terms in the differentially expressed gene list. Ranked list enrichment: enrichment of genes associated with a certain pathway towards the top of the ranked whole data. GO: gene ontology, BP: biological process, CC: cellular component, MF molecular function. KEGG: Kyoto Encyclopedia of Genes and Genomes. Annotated: number of genes associated with the given term. R: Reactome database. Significant: number of statistically significant associated with the given term. Expected: expected number of genes associated with the given term in the DE gene list. Gene ratio: ratio of number of genes in the DE list that overlap with genes associated with the given term, and the number of genes in the DE list which overlap with genes associated with all terms in the Reactome database. Bg ratio: ratio of the number of genes associated with the given term, and the total number of genes associated with Reactome terms

Ictal vs. healthy					
ID	DE list enrichment	Annotated	Significant	Expected	*p*-value
GO:1901700 BP	response to oxygen-containing compound	1347	45	10.86	2.60E-14
GO:0070851 MF	growth factor receptor binding	105	7	0.83	0.000019
		Gene ratio	Bg ratio		p-value
R-HSA-6783783	Interleukin-10 signaling	10/84	40/8821		2.60E-14
R-HSA-6785807	Interleukin-4 and Interleukin-13 signaling	11/84	98/8821		1.71E-09
R-HSA-179812	GRB2 events in EGFR signaling	3/84	11/8821		0.00013
	Ranked list enrichment	Annotated			p-value
GO:0070098 BP	chemokine-mediated signaling pathway	67			2.60E-14
KEGG 4080	Neuroactive ligand-receptor interaction	157			1.86E-14
KEGG 4740	Olfactory transduction	63			0.000817

In the interictal PBMC samples compared to healthy ones, cytokine and chemokine receptor binding, interleukin-10 (IL-10) signaling, as well as oxidative phosphorylation in the mitochondria were significantly affected.

In the ictal vs. interictal comparison, hormone and cytokine activity, oxidative phosphorylation, chemosensory receptors were implicated, among others.

In the ictal versus healthy comparison, IL-4, IL-10 and IL-13, as well as chemokine, growth factor and neuroactive ligand-receptor interactions were implicated.

**Fig. 4:**
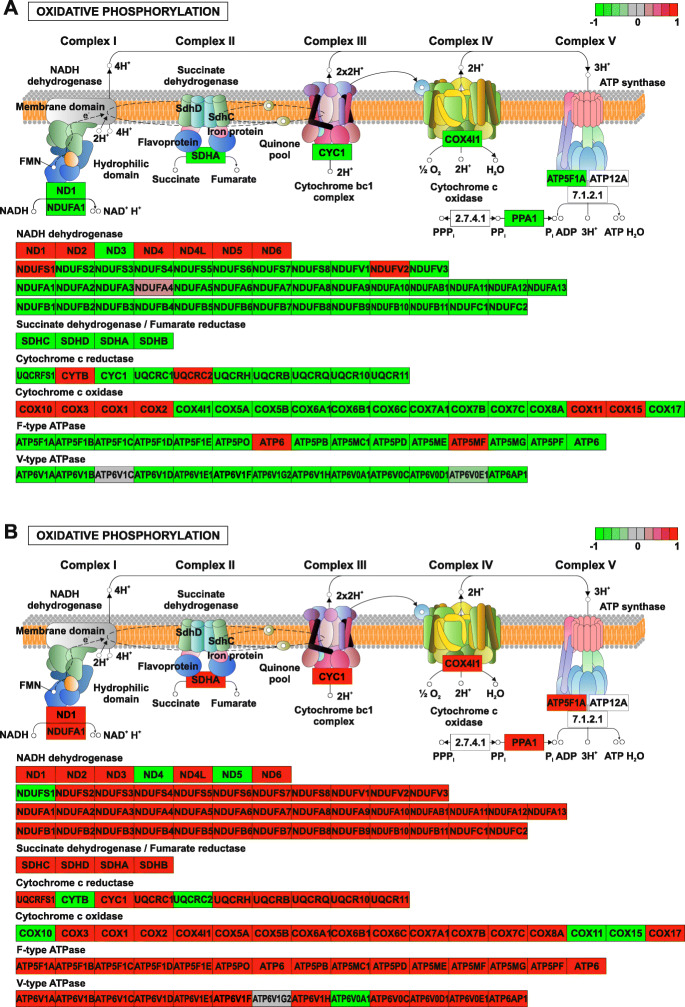
Oxidative phosphorylation was implicated as a significantly altered metabolic pathway based on the ranked list enrichment analysis of all genes whose transcripts were detected from PBMC samples. Results of the analysis between ictal vs. interictal (upper panel) and interictal vs. healthy (lower panel) samples are shown. All detected genes are mapped to the pathway map. Original figure was rendered by Pathview

Ranked list enrichment analysis of all genes statistically significantly implicated the metabolic pathway of oxidative phosphorylation (Tables 2, 3, 4) in the interictal PBMC samples when compared to the healthy control group (Fig. 4, upper panel) with a *p*-value of 8.82E-06, as well as in the ictal samples in comparison with the interictal group (Fig. 4, lower panel) with a *p*-value of 0.000845. Expression of most oxidative phosphorylation related genes on Fig. 4 were elevated in the interictal samples versus the healthy ones, while decreased in ictal samples during migraine attack when compared to the interictal groups. Expression of genes coding succinate dehydrogenase/fumarate reductase enzymes (SDHA, SDHB, SDHC, SDHD), for example, increased in the interictal samples versus the healthy ones, while decreased in ictal samples during migraine attack when compared to the interictal groups, albeit statistically non-significantly at the individual gene level.

### Metabolic alterations in the plasma of migraine patients

**Fig. 5:**
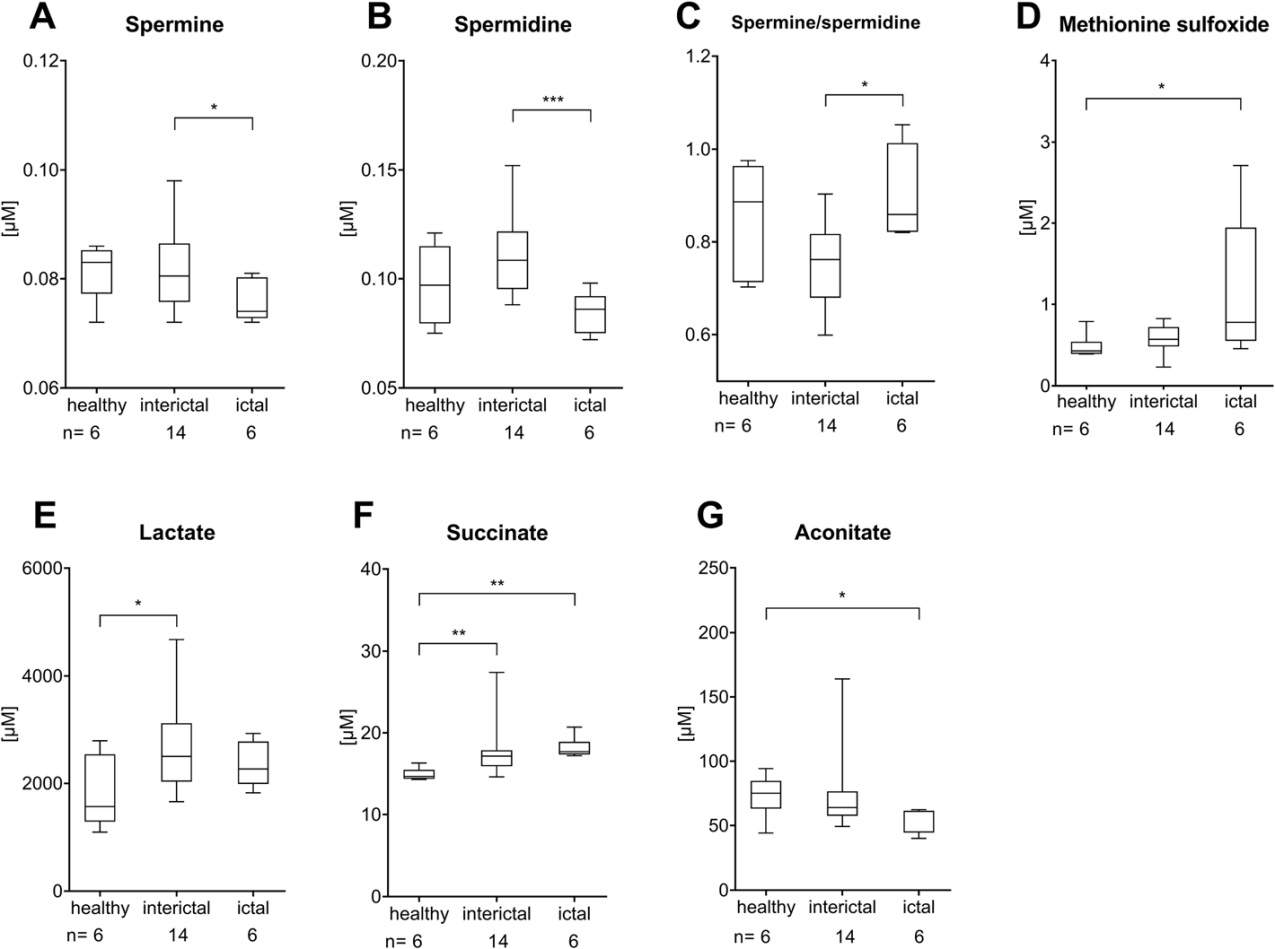
Differences in plasma metabolomic profiles of healthy controls and migraineurs (interictal and ictal) for different classes of compounds: (**A**, **B**, **C**) biogenic amines; (**D**) amino acid-related; and (**E**, **F**, **G**) carboxylic acids. Plasma concentrations of different metabolites are compared between groups and are considered significantly different when *p* ≤ 0.05. Asterisks denote significant differences (*p ≤ 0.05, **p ≤ 0.01, ***p ≤ 0.001), as analyzed by one-way analysis of variance (ANOVA) for A, B, C, E and Kruskal–Wallis test for **D**, **F**, **G** samples

Metabolomic measurements were performed on 14 interictal, 6 ictal and 6 healthy control plasma samples. During the migraine attack (ictal) spermine and spermidine levels were significantly reduced (Fig. 5A,B, *p*-values: 0.021 and < 0.001), in comparison to metabolite concentrations found in the samples from the attack-free period. Interestingly, the spermine/spermidine ratio was suppressed in migraineurs, but during headache the concentration ratio was restored to a healthy-like level (Fig. 5C, *p*-value: 0.014). Methionine sulfoxide levels significantly increased (Fig. 5D, *p*-values: 0.026) during the ictal phase, compared to the healthy group. Lactate and succinate levels were significantly elevated (Fig. 5E,F, *p*-values: 0.031 and 0.005) during the interictal phase when compared to healthy volunteers. Succinate concentration was also significantly higher (*p*-value: 0.0022) during the ictal phase compared to the healthy group, while aconitate was lower in the same comparison (Fig. 5G, *p*-value: 0.041).

## Discussion

This is the first transcriptome analysis of PBMCs isolated from interictal and ictal samples of migraine patients in comparison with healthy controls suggesting the importance of inflammatory pathways and the potential contribution of various cytokines to migraine susceptibility. In addition to the inflammatory pathways, our results suggest potential implication of mitochondrial dysfunction in migraine. Moreover, significant changes in some metabolites in the plasma also point to an alteration of mitochondrial electron transport chain or the citric acid cycle. There are two novel aspects of our analysis. On one hand, comparisons were made between healthy control samples to both the interictal and ictal samples of patients with the aim to identify both disease-specific and headache-specific alterations. On the other hand, instead of whole blood, we studied isolated mononuclear cells, the transcriptome of which had been reported to correlate better with the alterations in the brain [18]. Interestingly, significant gene expression and metabolite changes could be detected independently of headache episodes compared to healthy control samples which could be markers of migraine susceptibility, but not necessarily attack-specific.

A limitation of the study is that the RNA-based results are not confirmed by measuring the concentrations of the affected cytokines, but the results of the transcriptome analysis are in line with previous literature data which showed altered cytokine levels in migraine patients [33–36]. Our results showing immune pathway alterations are also in agreement with the findings of Gerring and coworkers’ next-generation RNA sequencing study of the whole blood of migraine patients [21]. A further limitation is that only the ictal vs. interictal comparison yielded gene results after controlling for the false discovery rate. Migraine can have a prevalence of 20% of the population thus it can include a heterogeneous patient pool. In this regard our examination can be considered rather exploratory in nature with key results to be confirmed in a later study with a larger number of participants. Some similar migraine studies, however, have included patients in similar order of magnitude [22]. Controlling for FDR indeed reduces the number of false positive results but in turn increases false negatives. The cost of the latter is missing out on important discoveries [37]. Not having sufficient results after correction for multiple comparison also hinders finding associated biological functions. In a similar setting, examined the whole blood of migraineurs, Kogelman and colleagues performed correction for multiple testing and found two genes to be significantly differentially expressed [22]. They ran, however, functional enrichment analysis on the full non-corrected DE gene list, as well as co-expression network analysis on 5000 genes which is a multiple of the number of DE genes in their study. We carried out functional analysis by two approaches. One was DE list enrichment where potential false positive results at the gene level are expected to be randomly distributed among associated biological functions. Thus, significant biological terms supported by several DE genes are less likely to be false positive hits themselves. Our other method was ranked list enrichment that is not limited to the DE genes, hence it can circumvent the challenges of multiple comparison. The analysis considers the ranked list of all genes whose transcripts were detected.

**Fig. 6:**
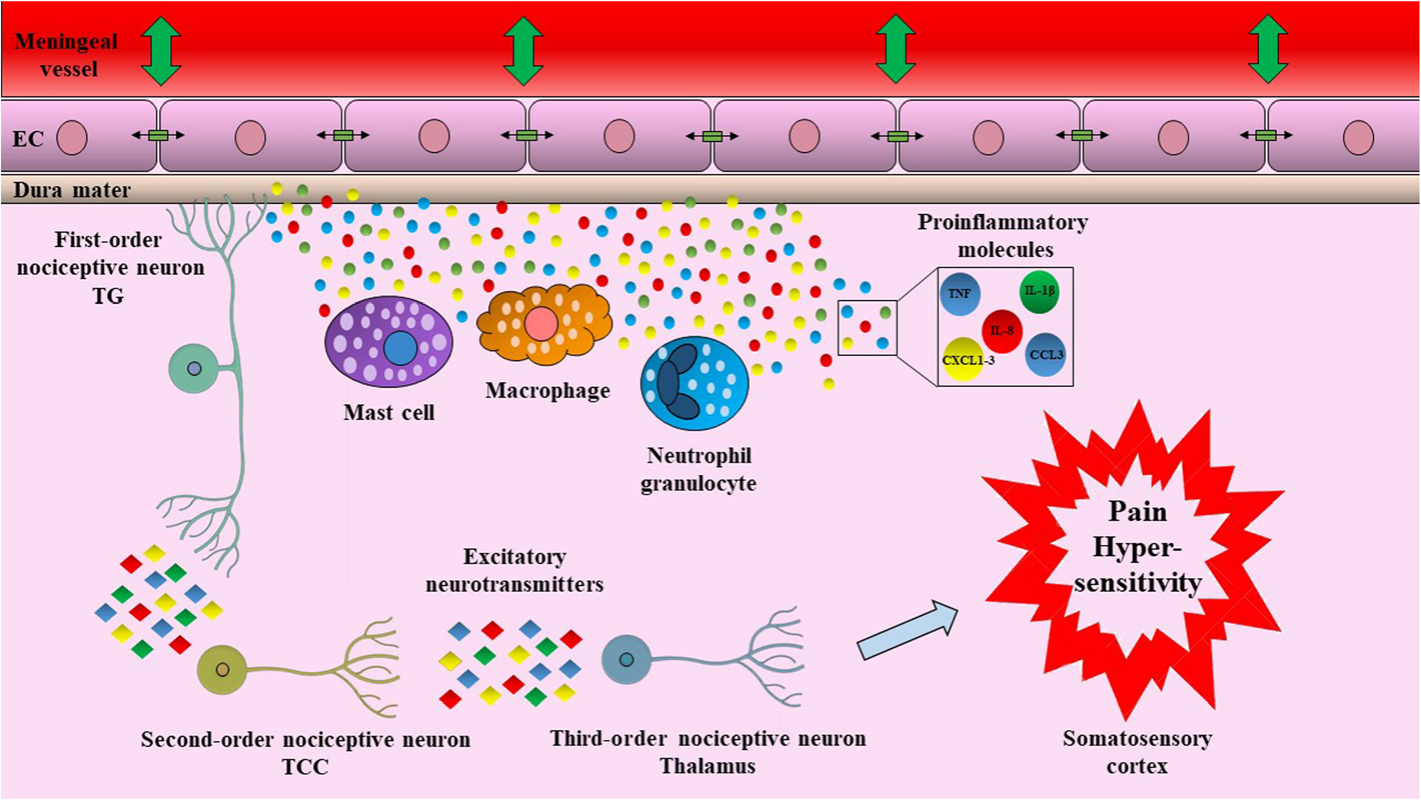
Hypothetical contribution of inflammatory cytokines in migraine headache. Activation of immune cells alters the trigeminovascular microenvironment via the release of inflammatory molecules, such as cytokines and chemokines. These molecules cause vasodilation of dural vasculature and influence tight junction between endothelial cells. Activated trigeminal neurons transmit signals for the higher brain regions, which result in pain and hypersensitivity. CCL3: C-C motif ligand 3, CXCL1: C-X-C motif chemokine ligand 1, CXCL2: C-X-C motif chemokine ligand 2, CXCL3: C-X-C motif chemokine ligand 3, EC: endothelial cell, IL-1β: interleukin-1β, IL-8: interleukin-8, TCC: trigeminocervical complex, TNF: tumor necrosis factor, TG: trigeminal ganglion

While the vascular and neuronal origin of migraine has been debated for a long time, it is clear that there is an inflammatory component in the generation of the headache. NSAIDs are partially effective to relieve the headache pointing to the contribution of prostaglandins to nociceptor sensitization. Activation of resident mast cells in the meninges and release of proinflammatory cytokines such as IL-1β, IL-6, TNF-α and several chemokines have been proposed to play major roles in the progression of migraine headache (Fig. 6) [38, 39]. Moreover, cytokine release by glial cells is also likely to contribute to migraine pathomechanism, as it was shown that cortical spreading depression can result in inflammosome activation in the brain parenchyma, as well [40]. In our study upregulation of several cytokines, as well as COX-2 were detected in PBMCs of migraine patients in both interictal and ictal samples compared to healthy controls pointing to a systemic change of immune functions. These data are in line with previous reports of elevated plasma levels of various proinflammatory cytokines like IL-1, IL-6 [33], TNFα [34], IL-8, CCL3 and CCL5 [41, 42] and C-reactive proteins (CRP) [43, 44]. Moreover, during attacks, the concentration of IL-1β, IL-6, IL-8, IL-10 and TNF-α were further increased [35, 36]. Cytokines are fundamental regulators of inflammatory and immune reactions, and several of them have been directly implicated in pain sensitization by acting both on peripheral nociceptive nerve terminals and sensory ganglia, as well as participating in central sensitization. The pro-nociceptive role of IL-1β or TNFα in both peripheral and central pain mechanisms is well characterized [45–48]. Their potential contribution to headaches was supported by data that application of IL-1β or TNFα on rat dura resulted in activation and sensitization of meningeal nociceptors [49, 50]. Other cytokines from our top DE gene list, such as CXCL1, 2 or 3 (all ligands of the CXCR2 receptor), have also been shown to be pro-nociceptive [51]. Intrathecal administration of CXCL1, 2 or 3 produced hyperalgesia in mice [52]. These chemokines have also been implicated in neuropathic pain [52–54] and nociceptive sensitization after traumatic brain injury [55, 56]. CXCL1 in the synovial fluid of osteoarthritis patients correlated with the severity of pain [57]. Furthermore, significantly upregulated genes in our study include amphiregulin and epiregulin, which are epidermal growth factor receptor (EGFR) ligands. These proteins have been implicated in tumor growth; however, a role in inflammation has also been described [58, 59]. Epiregulin, but not amphiregulin has been shown to be pro-nociceptive in mice, and EGFR inhibitors were analgesic in a variety of animal models of chronic pain [60]. Human data indicate that both amphiregulin and epiregulin expressions were higher in bone marrow-derived mononuclear cells of rheumatoid arthritis patients and increased expression of amphiregulin was also detected in PBMCs and synovial tissues [61].

Recently, the metabolic alterations in migraine have also been highlighted [62, 63] and a meta-analysis pointed to the importance of oxidative and nitrosative stress [64]. A possible link between mitochondrial dysfunction and neuroinflammation is the demonstration of NLRP3 inflammosome activation by mitochondrial reactive oxygen species which was postulated to participate in several CNS disorders including migraine [40]. Several lines of evidence demonstrate that in migraineurs there is an imbalance between energy requirement and supply of the brain [62, 63] and it has been hypothesized that the attack can be a consequence of an adaptive response to restore energy homeostasis. Impaired mitochondrial energy production has been detected in the brain and skeletal muscle of migraine patients during attacks, but also even interictally [65–68]. Moreover, it has also been raised that migraine triggers act as promoters of oxidative stress [69, 70] and various studies have consistently reported elevated levels of oxidative stress markers or a deficit of antioxidant mechanisms [62, 64]. Activities of various mitochondrial enzymes have been found to be altered in the platelets of migraine patients [71, 72].

In connection with the pathways identified with the PBMC transcriptome analysis, we have detected significant differences in several metabolites reflecting changes in mitochondrial function. These results provide a complementary viewpoint to a DE centric interpretation of our RNA-seq results. Lactate levels were increased in interictal, but not the ictal samples compared to the healthy plasma, while succinate levels were increased in both sets of migraine samples. Another intermediate metabolite of the citric acid cycle, aconitate was slightly decreased in parallel. Previous studies have already detected similar increases in plasma lactate and pyruvate levels [73], and it has also been reported that in migraine patients lactate levels rose higher upon exercise [74]. There are also data on increased lactate levels in some brain areas, although these were only detected in migraineurs with aura [62]. Abnormal “astrocyte-to-neuron lactate shuttle” has been considered to be behind the altered lactate metabolism, where astrocytes, through the end product of anaerobic glycolysis (lactate), provide energy to the activated neurons [75]. Regarding succinate, elevated levels were linked to a worse metabolic state in obese patients [76] and increased succinate concentrations have also been detected in the plasma of severely injured patients [77]. Also, succinate has been suggested to be a metabolic indicator of sepsis and mitochondrial dysfunction [78], with altered tissue concentrations under ischemia and inflammation [79–81]. However, novel data also indicate that succinate released for activated macrophages can act as a pro-inflammatory local mediator [80, 82], which could be another link between metabolic alterations in the plasma and inflammatory reactions. Other significantly changed metabolites include the polyamines spermine and spermidine and the oxidized metabolite of methionine, MetSO. Spermine and spermidine are involved in multiple cellular processes including the regulation of transcription and translation, alteration of ion channel and receptor function, and regulation of nitric oxide synthase. They act as scavengers of reactive oxygen radicals, thus represent important elements of normal mitochondrial functions. Polyamines also take part in protection from oxidative damage by stimulating the synthesis of superoxide dismutase, heat shock proteins and cell cycle regulators [83–89]. However, it has been described that over-production or over-intake of these polyamines might contribute to cellular damage by oxidative mechanisms. In the brain, they might play a role in gap junction permeability and neuronal hyperexcitability under pathological conditions. Interestingly, spermine also can regulate the activity of glutamate-receptors, TRPV1 channels, and glial Kir4.1 channels [90–94]. Methionine, due to exposure to reactive oxygen species (ROS), oxidizes to MetSO. This procedure can be reversed by the methionine sulfoxide reductase (Msr) [95]. Currently, a correlation has been suggested between the increment in MetSO plasma levels and Alzheimer’s disease progression [96]. Besides, altered Msr system function and MetSO accumulation have been proposed to be biomarkers of aging [97–99]. However, to our knowledge, this is the first study to link increased plasma levels of MetSO to migraine-related processes.

## Conclusions

In summary, our results suggest that enhanced immune cell activity and oxidative stress generation play important roles in migraine susceptibility and headache-generation. Detection of altered gene expressions and metabolite levels in the peripheral blood point to the systemic nature of the disease. Our study also indicates that drugs targeting cytokines or reducing oxidative stress might be valuable for migraine treatment or prophylaxis.

## Supplementary Information

**Additional file 1: Table S1.** Top 20 differentially expressed genes in PBMCs comparing interictal and healthy control samples. *Avg rank*: average rank of *p*-value and fold change ranks; *ID*: Ensembl gene identifier. **Table S2.** Top 20 differentially expressed genes in PBMCs comparing interictal and ictal samples. *Avg rank*: average rank of *p*-value and fold change ranks; *ID*: Ensembl gene identifier. **Table S3.** Top 20 differentially expressed genes in PBMCs comparing ictal and healthy control samples. *Avg rank*: average rank of *p*-value and fold change ranks; *ID*: Ensembl gene identifier.

## Data Availability

The RNA-Seq dataset supporting the conclusions of this article is available in the European Nucleotide Archive (https://www.ebi.ac.uk/ena), under accession number PRJEB40032.

## Abbreviations

PBMC: Peripheral blood mononuclear cells
CGRP: Calcitonin gene-related peptide
PACAP: Pituitary adenylate cyclase activating polypeptide
GWAS: Genome-wide association studies
EDTA: Ethylenediaminetetraacetic acid
PBS: Phosphate-buffered saline
FC: Fold change
FDR: False discovery rate
GO: Gene ontology
KEGG: Kyoto encyclopedia of genes and genomes
DE: Differentially expressed
PITC: Phenyl isothiocyanate
LC-MS/MS: Liquid chromatography-mass spectrometry
FIA-MS/MS: Flow injection analysis-tandem mass spectrometry
QA: Quality assurance
QC: Quality control
ANOVA: Analysis of variance
BMI: Body mass index
IL: Interleukin
PTGS2: Prostaglandin-endoperoxide synthase 2
COX2: Cyclooxygenase 2
TNF: Tumor necrosis factor
CRP: C-reactive protein
EGFR: Epidermal growth factor receptor
ROS: Reactive oxygen species
