# Supplementary material for: Identification of disease- and headache-specific mediators and pathways in migraine using blood transcriptomic and metabolomic analysis
Source: J Headache Pain. 2021 Oct 6;22(1):117. doi: 10.1186/s10194-021-01285-9 (PMC8493693; doi:10.1186/s10194-021-01285-9)
Supplement: Supplementary file 1 — Additional file 1: Table S1. Top 20 differentially expressed genes in PBMCs comparing interictal and healthy control samples. Avg rank: average rank of p-value and fold change ranks; ID: Ensembl gene identifier. Table S2. Top 20 differentially expressed genes in PBMCs comparing interictal and ictal samples. Avg rank: average rank of p-value and fold change ranks; ID: Ensembl gene identifier. Table S3. Top 20 differentially expressed genes in PBMCs comparing ictal and healthy control samples. Avg rank: average rank of p-value and fold change ranks; ID: Ensembl gene identifier. [file 10194_2021_1285_MOESM1_ESM.docx]

**Supplementary Materials**

| **Interictal vs. Healthy** | | | | | | |
| --- | --- | --- | --- | --- | --- | --- |
| avgRank | ID | FC | P-Value | adj.  P-value | Description | geneName |
| 1 | ENSG00000125538 | 13.6 | 1.27E-04 | 0.98947 | interleukin 1 beta | IL1B |
| 2 | ENSG00000073756 | 7.8 | 4.85E-04 | 1 | prostaglandin-endoperoxide synthase 2 (cyclooxygenase 2) | PTGS2 (COX2) |
| 3 | ENSG00000169429 | 7.0 | 9.72E-04 | 1 | C-X-C motif chemokine ligand 8 | IL8 |
| 4 | ENSG00000232810 | 7.0 | 1.19E-03 | 1 | tumor necrosis factor | TNF |
| 5 | ENSG00000163739 | 8.7 | 2.80E-03 | 1 | C-X-C motif chemokine ligand 1 | CXCL1 |
| 6 | ENSG00000165685 | 5.3 | 3.84E-04 | 1 | transmembrane protein 52B | TMEM52B |
| 7 | ENSG00000205021 | 8.5 | 2.91E-03 | 1 | C-C Motif Chemokine Ligand 3 Like 1 | CCL3L1 |
| 8 | ENSG00000205595 | 7.0 | 2.55E-03 | 1 | amphiregulin B | AREGB |
| 9 | ENSG00000120738 | 6.1 | 1.56E-03 | 1 | early growth response 1 | EGR1 |
| 10 | ENSG00000124882 | 8.4 | 3.62E-03 | 1 | epiregulin | EREG |
| 11 | ENSG00000087074 | 3.8 | 1.71E-04 | 0.98947 | protein phosphatase 1 regulatory subunit 15A | PPP1R15A |
| 12 | ENSG00000112149 | 5.7 | 2.00E-03 | 1 | CD83 molecule | CD83 |
| 13 | ENSG00000140379 | 3.8 | 1.83E-04 | 0.98947 | BCL2 related protein A1 | BCL2A1 |
| 14 | ENSG00000109321 | 6.6 | 2.68E-03 | 1 | amphiregulin | AREG |
| 15 | ENSG00000163661 | 8.3 | 4.17E-03 | 1 | pentraxin 3 | PTX3 |
| 16 | ENSG00000124107 | 4.6 | 1.15E-03 | 1 | secretory leukocyte peptidase inhibitor | SLPI |
| 17 | ENSG00000129277 | 4.8 | 1.27E-03 | 1 | C-C Motif Chemokine Ligand 4 | CCL4 |
| 18 | ENSG00000123689 | 10.6 | 5.67E-03 | 1 | G0/G1 switch 2 | G0S2 |
| 19 | ENSG00000256515 | 8.8 | 5.54E-03 | 1 | C-C Motif Chemokine Ligand 3 Like 3 | CCL3L3 |
| 20 | ENSG00000222040 | 9.2 | 6.54E-03 | 1 | Adrenoceptor Alpha 2B | ADRA2B |

**Table S1.** Top 20 differentially expressed genes in PBMCs comparing interictal and healthy control samples. *Avg rank*: average rank of p-value and fold change ranks; *ID*: Ensembl gene identifier;

| **Ictal vs. Interictal** | | | | | | |
| --- | --- | --- | --- | --- | --- | --- |
| avgRank | ID | FC | P-Value | adj.  P-value | Description | geneName |
| 1 | ENSG00000179172 | -3.2 | 1.09E-07 | 0.001766 | heterogeneous nuclear ribonucleoprotein C like 1 | HNRNPCL1 |
| 2 | ENSG00000267908 | -2.8 | 5.96E-04 | 1 | zinc finger and SCAN domain containing 5D pseudogene | ZSCAN5D |
| 3 | ENSG00000268154 | -3.0 | 3.48E-03 | 1 | Metazoan signal recognition particle RNA | AL591806.1 |
| 4 | ENSG00000248874 | 3.4 | 4.86E-03 | 1 | chromosome 5 open reading frame 17 (putative) | C5orf17 |
| 5 | ENSG00000229937 | 2.6 | 1.60E-03 | 1 | phosphoribosyl pyrophosphate synthetase 1 like 1 | PRPS1L1 |
| 6 | ENSG00000180389 | -2.5 | 2.05E-04 | 1 | ATP synthase F1 subunit epsilon pseudogene 2 | ATP5EP2 |
| 7 | ENSG00000255582 | 3.1 | 4.90E-03 | 1 | olfactory receptor family 10 subfamily G member 2 | OR10G2 |
| 8 | ENSG00000133136 | -2.5 | 1.47E-03 | 1 | G protein subunit gamma 5 pseudogene 2 | GNG5P2 |
| 9 | ENSG00000120156 | 2.7 | 4.55E-03 | 1 | TEK receptor tyrosine kinase | TEK |
| 10 | ENSG00000125207 | -2.6 | 4.47E-03 | 1 | piwi like RNA-mediated gene silencing 1 | PIWIL1 |
| 11 | ENSG00000180535 | 2.5 | 4.17E-03 | 1 | basic helix-loop-helix family member a15 | BHLHA15 |
| 12 | ENSG00000169213 | -2.6 | 7.03E-03 | 1 | RAB3B, member RAS oncogene family | RAB3B |
| 13 | ENSG00000176994 | 2.4 | 4.29E-03 | 1 | SMCR8-C9orf72 complex subunit | SMCR8 |
| 14 | ENSG00000160838 | -2.6 | 6.35E-03 | 1 | leucine rich repeat containing 71 | LRRC71 |
| 15 | ENSG00000235478 | -2.4 | 5.08E-03 | 1 | long intergenic non-protein coding RNA 1664 | AC006946.15 |
| 16 | ENSG00000069812 | -2.6 | 1.10E-02 | 1 | hes family bHLH transcription factor 2 | HES2 |
| 17 | ENSG00000016402 | -2.7 | 1.25E-02 | 1 | interleukin 20 receptor subunit alpha | IL20RA |
| 18 | ENSG00000070031 | -2.4 | 5.75E-03 | 1 | secretin | SCT |
| 19 | ENSG00000254788 | 2.4 | 7.64E-03 | 1 | CKLF-CMTM1 readthrough | CKLF-CMTM1 |
| 20 | ENSG00000153976 | 2.2 | 2.65E-04 | 1 | heparan sulfate-glucosamine 3-sulfotransferase 3A1 | HS3ST3A1 |

**Table S2.** Top 20 differentially expressed genes in PBMCs comparing interictal and ictal samples. *Avg rank*: average rank of p-value and fold change ranks; *ID*: Ensembl gene identifier;

| **Ictal vs. Healthy** | | | | | | |
| --- | --- | --- | --- | --- | --- | --- |
| avgRank | ID | FC | P-Value | adj.  P-value | Description | geneName |
| 1 | ENSG00000125538 | 21.7 | 1.14E-04 | 1 | interleukin 1 beta | IL1B |
| 2 | ENSG00000073756 | 10.8 | 3.27E-04 | 1 | prostaglandin-endoperoxide synthase 2 (cyclooxygenase 2) | PTGS2 (COX2) |
| 3 | ENSG00000205021 | 13.9 | 9.44E-04 | 1 | C-C Motif Chemokine Ligand 3 Like 1 | CCL3L1 |
| 4 | ENSG00000232810 | 10.8 | 5.02E-04 | 1 | tumor necrosis factor | TNF |
| 5 | ENSG00000124882 | 11.8 | 1.33E-03 | 1 | epiregulin | EREG |
| 6 | ENSG00000256515 | 14.5 | 1.72E-03 | 1 | C-C Motif Chemokine Ligand 3 Like 3 | CCL3L3 |
| 7 | ENSG00000197262 | 8.4 | 1.18E-03 | 1 | C-C Motif Chemokine Ligand 4 Like 2 | CCL4L2 |
| 8 | ENSG00000006075 | 12.6 | 1.83E-03 | 1 | C-C Motif Chemokine Ligand 3 | CCL3 |
| 9 | ENSG00000169429 | 9.2 | 1.43E-03 | 1 | C-X-C motif chemokine ligand 8 | IL8 |
| 10 | ENSG00000205020 | 8.2 | 1.35E-03 | 1 | C-C Motif Chemokine Ligand 4 Like 1 | CCL4L1 |
| 11 | ENSG00000163734 | 9.4 | 1.80E-03 | 1 | C-X-C motif chemokine ligand 3 | CXCL3 |
| 12 | ENSG00000112149 | 7.3 | 1.40E-03 | 1 | CD83 molecule | CD83 |
| 13 | ENSG00000081041 | 12.1 | 2.87E-03 | 1 | C-X-C motif chemokine ligand 2 | CXCL2 |
| 14 | ENSG00000129277 | 6.4 | 1.01E-03 | 1 | C-C Motif Chemokine Ligand 4 | CCL4 |
| 15 | ENSG00000165685 | 5.7 | 5.72E-04 | 1 | transmembrane protein 52B | TMEM52B |
| 16 | ENSG00000163739 | 9.7 | 2.69E-03 | 1 | C-X-C motif chemokine ligand 1 | CXCL1 |
| 17 | ENSG00000173391 | 8.4 | 3.10E-03 | 1 | oxidized low density lipoprotein receptor 1 | OLR1 |
| 18 | ENSG00000123689 | 13.0 | 5.01E-03 | 1 | G0/G1 switch 2 | G0S2 |
| 19 | ENSG00000120738 | 7.2 | 2.05E-03 | 1 | early growth response 1 | EGR1 |
| 20 | ENSG00000227921 | 7.3 | 2.18E-03 | 1 | putative uncharacterized protein | AL353791.1 |

**Table S3.** Top 20 differentially expressed genes in PBMCs comparing ictal and healthy control samples. *Avg rank*: average rank of p-value and fold change ranks; *ID*: Ensembl gene identifier;
